# The Δ*F/F*_m_′-guided supply of nitrogen in culture medium facilitates sustainable production of TAG in *Nannochloropsis oceanica* IMET1

**DOI:** 10.1186/s13068-018-1168-y

**Published:** 2018-06-20

**Authors:** Jiao Liu, Changhong Yao, Yingying Meng, Xupeng Cao, Peichun Wu, Song Xue

**Affiliations:** 1 Marine Bioengineering Group, Dalian Institute of Chemical Physics, Chinese Academy of Sciences, Dalian, 16023 China; 2 University of Chinese Academy of Sciences, Beijing, 100049 China; 3 Department of Pharmaceutical & Biological Engineering, School of Chemical Engineering, Sichuan University, Chengdu, 610065 China; 4 College of Chemistry, Chemical Engineering and Materials Science, Shandong Normal University, Jinan, 250014 China

**Keywords:** Semi-continuous cultivation, Δ*F/F*_m_*′* monitoring, Nitrogen stress index, Microalgae

## Abstract

**Background:**

Triacylglycerol (TAG) from photosynthetic microalgae is a sustainable feedstock for biodiesel production. Physiological stress triggers microalgal TAG accumulation. However excessive physiological stress will impair the photosynthesis system seriously thus decreasing TAG productivity because of the low biomass production. Hence, it is critical to quantitatively and timely monitor the degree of the stress while the microalgal cells growing so that the optimal TAG productivity can be obtained.

**Results:**

The lack of an on-line monitored indicator has limited our ability to gain knowledge of cellular “health status” information regarding high TAG productivity. Therefore, to monitor the degree of nitrogen stress of the cells, we investigated the correlation between the photosynthetic system II (PS II) quantum yield and the degree of stress based on the high relevancy between photosynthetic reduction and nitrogen stress-induced TAG accumulation in microalgal cells. Δ*F/F*_m_′, which is the chlorophyll fluorescence parameter that reflects the effective capability of PS II, was identified to be a critical factor to indicate the degree of stress of the cells. In addition, the concept of a nitrogen stress index has been defined to quantify the degree of stress. Based on this index and by monitoring Δ*F/F*_m_′ and guiding the supply of nitrogen in culture medium to maintain a stable degree of stress, a stable and efficient semi-continuous process for TAG production has been established.

**Conclusion:**

The results indicate that the semi-continuous cultivation process with a controlled degree of stress by monitoring the Δ*F/F*_m_′ indicator will have a significant impact on microalgal TAG production, especially for the outdoor controllable cultivation of microalgae on a large scale.

## Background

Photosynthetic microalgae have received increasing attention as the most promising biofuel feedstock as humans are facing increasing problems related to climate and fossil energy [1–3]. Microalgae can use light energy and CO_2_ to produce energy-storage compounds such as triacylglycerol (TAG), which is the precursor of biodiesel [4]. Physiological stress is usually applied to microalgal cultures to trigger TAG accumulation. When confronted with physiological stress, such as nitrogen stress (N-stress), microalgal cells make adjustments such as enhancing the energy-storage compounds (e.g., TAG) accumulation, to acclimate to unfavourable conditions. However, the physiological stress affects both photosynthesis and C-storage mechanism. When under excessive physiological stress, the photosynthetic efficiency progressively decreases and the cell growth is significantly diminished. Therefore, it is quite challenging to achieve maximum TAG productivity by balancing the TAG content and high productivity of microalgal biomass because TAG production largely relies on stress conditions such as nitrogen stress (N-stress) and high irradiance etc. [5].

N-stress is the most effective means to trigger TAG accumulation [4, 6, 7]. When subjected to N-stress, in addition to enhanced TAG accumulation, protein synthesis in microalgal cells is affected as well. Protein synthesis in microalgae is immediately suppressed upon nitrogen shortage, which mostly hinders the protein turnover of photosynthetic apparatus, especially the photosystem (PS) II D1 reaction centre protein [8]. This will lead to a decline in the photosynthetic electron transport rate (ETR) and, consequently, a reduction in photochemical energy conversion [8, 9]. Moreover, limited nitrogen supply causes impairment of photosynthetic CO_2_ fixation by degradation of ribulose-1,5-bisphosphate (RuBP) carboxylase/oxygenase (Rubisco) for the recycling of nitrogen. Limitation of CO_2_ fixation then decreases the consumption of ATP and NADPH and leads to an excess of NADPH and electrons [8]. The sufficient supply of NADPH is essential for TAG accumulation [10], whereas excessive electrons will lead to the formation of reactive oxygen species (ROS), which exposes microalgae to oxidative stress and is also believed to be a signal trigger for TAG formation [11–13]. In addition, the remodelling of the photosynthetic membranes caused by N-stress also contributes to a considerable fraction of TAG production by providing a fatty acid acyl moiety [5, 14, 15]. Therefore, it is closely linked to photosynthetic reduction and N-stress-induced TAG accumulation in microalgal cells. Therefore, it is very important to make systematic research on it.

Photosynthesis is a coordinated physiological process that exclusively provides both energy and the material foundation for photoautotrophic microalgae [16]. Therefore, it could be considered the most important cellular metabolism in algae. With no exception, photosynthesis provides the energy as well as fixes the carbon used for TAG synthesis in photoautotrophic microalgae. However, as stated above, TAG accumulation is inevitably accompanied by photosynthetic reduction under excessive stress in photoautotrophic oleaginous microalgae, which means that excessive stress could cause inhibition of growth and reduce biomass and overall TAG yield. Optimal TAG productivity can be achieved only if the photosynthetic performance is properly maintained. Therefore, quantitative and timely monitoring of the stress status of the subjected microalgal cells is vital so that the optimal TAG productivity can be attained in time. Because of the tight relationship between photosynthesis and TAG synthesis, monitoring of the photosynthetic performance should be a plausible method.

Chlorophyll fluorescence analysis is a powerful tool for the study of photosynthesis in both plants and algae [17–19]. It allows a non-invasive and nearly instantaneous measurement of performance in photosynthetic light capture and electron transport [17]. The PS II quantum yield, which is a chlorophyll fluorescence parameter that measures the proportion of the light absorbed by chlorophyll related to PS II and is used in photochemistry, provides an estimation of the linear ETR and hence the PS II performance [18, 19]. It is widely used as the indicator to assess nutrient limitations in situ in microalgae [9, 20, 21]. However, to the best of our knowledge, little research has been conducted hitherto to apply this fluorescence parameter for the control of TAG production. In our previous work, a decreased PS II quantum yield was found to be highly related to the degree of N-stress. In both *Tetraselmis subcordiformis* and *Isochrysis zhangjiangensis*, critical values of chlorophyll fluorescence existed where the maximum productivities of energy storage substances, such as carbohydrates, were obtained [22, 23]. Therefore, in this work, we proposed the use of this fluorescence parameter as an N-stress indicator to monitor the stress degree and TAG production during cultivation by controlling the nitrogen supply strategy. To obtain an efficient and stable production of lipid from *Nannochloropsis oceanica* IMET1, we established a reproducible TAG production process by monitoring photosynthetic activity to maintain a constant degree of N-stress under semi-continuous cultivation.

## Methods

### Strain and Cultivation conditions

*Nannochloropsis oceanica* IMET1 from the University of Maryland Biotechnology Institute was cultivated in seawater with modified F/2 medium at the ambient temperature of 25 ± 1 °C. In the present study, a 500-mL bubble column bioreactor (5 cm in diameter) was used with the air flow at 100 mL/min (2% CO_2_, v/v) filtered by a 0.22-μm membrane as described by Pan et al. [24]. Additionally, 140 μmol/m^2^ s cool white fluorescent lights were provided from one side of the bioreactor under a 14-h/10-h light/dark cycle. After pre-culture in the bioreactor with sufficient nutrient elements until the cells reached exponential growth phase, the cells were inoculated into new bioreactors at an initial biomass concentration of ~ 0.18 mg/mL (dry weight, DW).

For the batch culture, the initial N amount was 15 mg/L in the medium, and no additional N was added into the medium until harvest. For the semi-continuous cultivation, the inoculum was the N-stressed cells from the batch. During cultivation, the fluorescence parameter Δ*F/F*_m_′ was monitored. During the semi-continuous, Δ*F/F*_m_′ was used as an indicator of the degree of N-stress. When Δ*F/F*_m_′ reached the predetermined level, which was determined in batch cultivation as the monitoring point, harvest and dilution were conducted to start a new cycle of cultivation. During the dilution, the relative amount of fresh medium replenished to the system was defined as the “renewal rate”. For each cycle during the same semi-continuous cultivation process, the initial biomass concentration and supplementary N amount were the same.

### Measurements

Microalgal pellets from centrifugation were washed twice with 0.5 M NH_4_HCO_3_ and were then dried at 60 °C to achieve a constant weight. The DW was the difference between the final weight and the weight of the empty tube as described by Zhu et al. [25]. According to Chi et al. [26], the nitrate concentrations in the medium were measured using a UV/VIS spectrophotometer with a pre-drawn standard curve of the nitrate absorption of light. The light intensity was measured by an Optometer P9710 using a photosynthetically active radiation detector (Gigahertz Optik Corporation, Germany). The nitrogen quota of the biomass was determined using an elemental analysis instrument (Vario EL cube, Elementar Analysensysteme GmbH Germany). The fluorescence parameter Δ*F/F*_m_′ was measured using a chlorophyll fluorometer (Water-PAM WALZ, Germany) by the method described by Yao et al. [22]. After applying a saturating light pulse to the light-adapted cells, *F*_m_′ and *F′* were obtained, and *F*_m_′ refers to the maximum fluorescence at the light-adapted state. The effective photosynthesis capability of PS II, Δ*F/F*_m_′, was calculated according to Eq. (1).

1 $$ \Delta F/F_{\text{m}}^{\prime} = (F_{\text{m}}^{\prime} - F^{\prime} )/F_{\text{m}}^{\prime}$$

The TAG content was calculated using the method developed by Wang and Shen and based on the concept of characteristic fatty acid, which provided a way to quantify TAGs in microalgae precisely by immediate transesterification from the wet biomass and required smaller samples than conventional methods [27, 28]. Shen et al. identified EPA as the characteristic fatty acid of *N. oceanica* and confirmed the highly linear correlation between the TAG content and the relative percentage content of EPA obtained by normalizing the total fatty acids (regression equation: *y* = 36.737 − 1.212*x*, where *R*^2^ = 0.97, *y* is the TAG content, and *x* is the relative percent content of EPA). The fatty acid composition was determined by gas chromatography after transesterification and according to Liu et al. [29]. TAG productivity was calculated by Eq. (2), where DW_*i*_ and *C*_TAG−*i*_ represent the DW and TAG content at the end of cycle *i*, and the symbols with the subscript of cycle 0 represent the corresponding data of the initial state of cycle *i*.

2 $$ P_{i} = ({\text{DW}}_{i} \times C_{{\text{TAG}} - i} - {\text{DW}}_{i0} \times C_{{\text{TAG}} - i0} )/t $$

## Results and discussion

### Verification of Δ*F/F*_m_′ as an N-stress indicator for TAG production

**Fig. 1:**
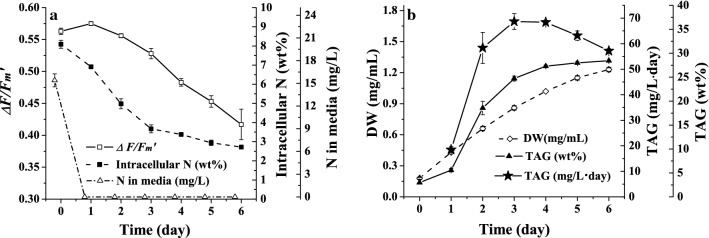
N-limited cultivation of *N. oceanica* IMET1

The N-stress indicator should be automatically and rapidly measured. It should be sensitive to N-stress, including the content of intracellular N and other parameters of physiology or biochemistry, such as the metabolite content, elemental content, photosynthesis activity, and respiration rate. Therefore, during cell growth and TAG accumulation under N-stress, the physiological characteristics and cellular biochemical properties were investigated including the DW, TAG content, intracellular N content and content of N in the medium and the chlorophyll fluorescence parameter Δ*F/F*_m_′. As shown in Fig. 1a, the N in the medium was completely consumed in one day, and the intracellular N decreased over time from 8.09 to 2.72%, which indicated that the cells became N-stressed from day 1. Another parameter, Δ*F/F*_m_′, which reflects the effective photosynthesis capability of PS II, decreased consistently with changes in intracellular N from 0.56 to 0.42. As shown in Fig. 1b, the TAG content increased rapidly in the first 4 days, from 3 to 27.3%, and increased slowly after day 5, from 28.0 to 28.4%; the growth rate of DW decreased over time from 0.17 mg/mL day at day 4 to 0.13 mg/mL day at day 5 and further to 0.08 mg/mL day at day 6. The daily TAG productivity shown in Fig. 1b reached the highest level at day 4 and then obviously decreased. Because the TAG productivity was related to the DW and TAG content, the increase in the DW and TAG content became slower and thus led to the decrease in TAG productivity after day 4. This indicates that the improvement in TAG content by strengthening N-stress will conversely lead to the slowdown or even the stoppage of cell growth, which results in lower TAG productivity.

When comparing all of the parameters, intracellular N is a critical parameter to trigger the initiation of TAG accumulation. There were studies that made efforts on the determining the proper N-stress and emphasized the importance of accurate control of intracellular N when to optimize the lipid productivity [30, 31]. However, the lack of a fast detection method for intracellular N limits its application as an online indicator to control microalgal cells at a proper degree of N-stress and to balance cell growth and TAG accumulation. Among the measured parameters, Δ*F/F*_m_′ is a promising indicator of N-stress in microalgae due to the high correlation between intracellular N and Δ*F/F*_m_′. By investigating the growth of *N. oceanica* IMET1 under N-stress, it was found that during the period of day 3 to day 6 when the highest TAG productivity under N-stress was obtained, there was a high correlation between intracellular N and Δ*F/F*_m_′. The correlation coefficient between intracellular N and Δ*F/F*_m_′ reached 0.97 (*y* = 0.1083*x* + 0.1258, where *R*^2^ = 0.9713, *y* refers to Δ*F/F*_m_′, and *x* refers to intracellular N). In the other species of microalgae, a correlation also exists between intracellular N and Δ*F/F*_m_′. For *T. subcordiformis* and *I. zhangjiangensis*, the correlations were *y* = 0.0741*x* + 0.151 (*R*^2^ = 0.9779) and *y* = 0.168*x* + 0.1597 (*R*^2^ = 0.9302), respectively (unpublished data). In short, it is not a special case where the Δ*F/F*_m_′ and intracellular N synchronously change while cells are under the N-stress condition. The great advantages of Δ*F/F*_m_′ are its characteristic of in situ and rapid measurement. In addition, the photosystem (whose activity Δ*F/F*_m_′ reflects) plays the role of the hub in microalgal metabolism. Therefore, we propose monitoring the degree of N-stress using Δ*F/F*_m_′ as an on-line indicator and then making the unsteady status of high TAG productivity reproducible to realize continuous TAG production.

### Determination of the proper degree of N-stress of microalgal cells for TAG production

High TAG productivity is highly related to the degree of N-stress, and determination of the proper degree of N-stress of microalgal cells is required. The proper degree of N-stress could not only induce TAG accumulation but also maintain the cells at a relatively high photosynthesis capability for growth and thus ensure the balance of the contradiction between TAG accumulation and fast growth. From day 3 to day 5, as shown in Fig. 1, both the TAG productivity and Δ*F/F*_m_′ stayed at a relatively high level, above 63 mg/mL day and 0.45, which was 78% of the maximum value of 0.58, respectively, as shown in Fig. 1b. Therefore, it could be concluded that from day 3 to day 5 the cells would reach the status with the proper degree of N-stress. To determine the proper degree of N-stress as the monitoring point and to realize semi-continuous cultivation under the constant N-stress condition and investigate the recovery of N-stressed cells after N replenishment, three batches of cultivation were conducted using N-stressed cells obtained at day 3, day 4 and day 5 as inoculum (day3-cell, day4-cell and day5-cell). Before recultivating the N-stressed cells, a new concept of the N-stress Index (NSI) was defined to quantify the degree of N-stress, which is never named. So far, the degree of stress has never been maintained by controlling any parameter. The NSI is calculated based on the intracellular N according to Eq. (3), where *N*_max_ is the maximum intracellular N content of the cells under N-replete conditions, *N*_min_ refers to the minimum intracellular N for the cells to only keep alive subjected to severe N-stress for a long time, and *N*_*i*_ is the intracellular N of the cells at day i. Therefore, NSI ranges from 0 to 1 and a greater value of NSI indicates a higher degree of N-stress of the cells. Then, the NSIs of the day3-cell, day4-cell and day5-cell were 0.62, 0.72 and 0.81, respectively, and represent aggravated N-stress of the cells.

3 $$ {\text{NSI}}_{i} = (N_{{\rm max} } - N_{i} )/(N_{\rm max} - N_{\rm min} ) $$

**Fig. 2:**
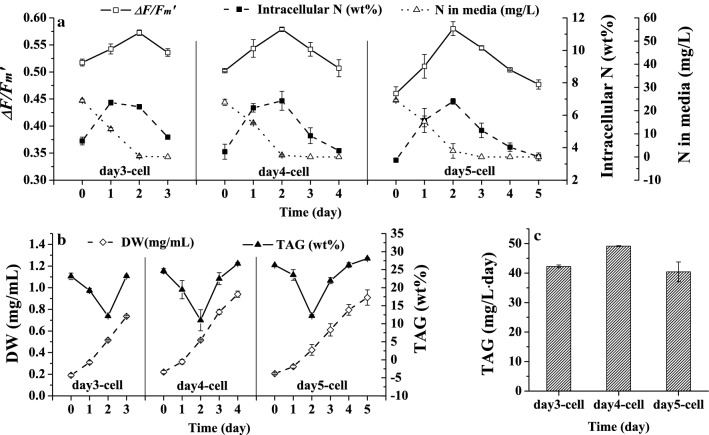
N-limited cultivation of different N-stressed cells

**Fig. 3:**
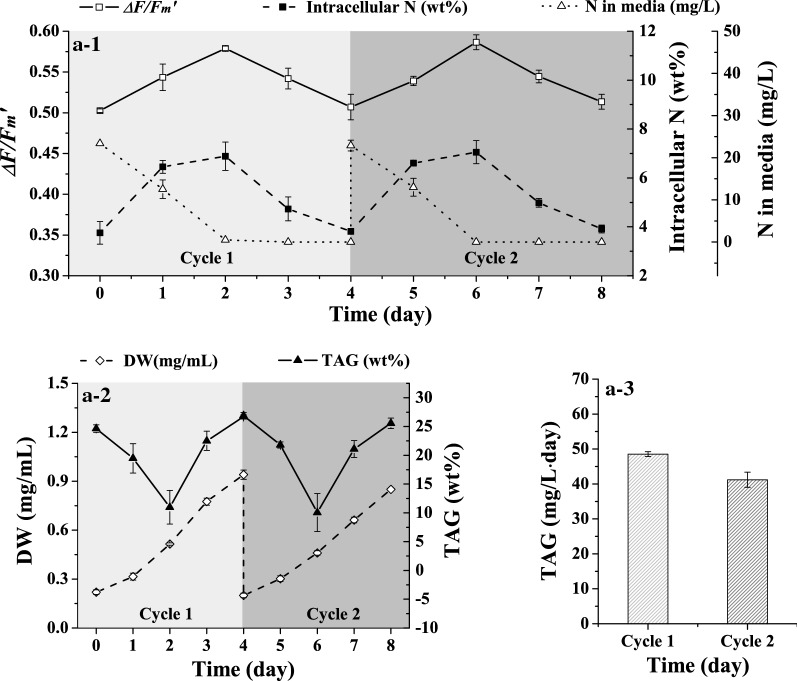
Semi-continuous cultivation of day4-cell

During the recultivation, Δ*F/F*_m_′ was used as an N-stress indicator in situ. The cultivation of the day4-cell is used as an example to describe in detail the actual operation. The Δ*F/F*_m_′ of the day4-cell was 0.503 at the inoculation, and this value was set as the critical point of monitoring. After supplying N into the medium and culturing for a few days, when the Δ*F/F*_m_′ reached the same level as the preset critical point at inoculation, the cultivation was ended. Specifically, as shown in Fig. 2a, after 4 days of cultivation, when the Δ*F/F*_m_′ reached 0.507, which was the same level as the initial Δ*F/F*_m_′, that is, 0.503 for the day4-cell, the cultivation was ended. The operations of the day3-cell and day5-cell were the same as those of the day4-cell, and actual changes in Δ*F/F*_m_′ can be seen in Fig. 2a. The daily intracellular N was also measured and depicted in Fig. 2a. Moreover, the TAG content also reached the expected level, as shown in Fig. 2b. The above results confirmed that it is feasible to reproduce cells at a specified status to produce TAG by monitoring Δ*F/F*_m_′. In addition, from the results of the TAG productivity obtained by cultivation of the 3 types of N-stressed cells shown in Fig. 3c, it was obvious that the TAG productivity of the day4-cell was higher than those of the other two types of cells and reached 49 mg/mL day. For the day3-cell, even though the Δ*F/F*_m_′ was higher and indicated a faster growth rate than the other two kinds of cells, the TAG accumulation was lower because the cells encountered a lower degree of N-stress, which ultimately resulted in less TAG productivity than the day4-cell. The result of the day3-cell indicated that the NSI of 0.62 was not large enough. Different from the day3-cell, a lower growth rate of day5-cells led to a lower TAG productivity. In contrast to the day3-cell, day5-cells encountered excessive N-stress with a large NSI of 0.81, which resulted in too much impairment of the cell growth. Therefore, according to the above results, the day4-cell experienced the proper degree of N-stress with an NSI of 0.72, and the corresponding Δ*F/F*_m_′ of 0.503 could be considered the critical monitoring point.

### Stable reproduction of TAG under semi-continuous cultivation by monitoring Δ*F/F*_m_*′*

**Fig. 4:**
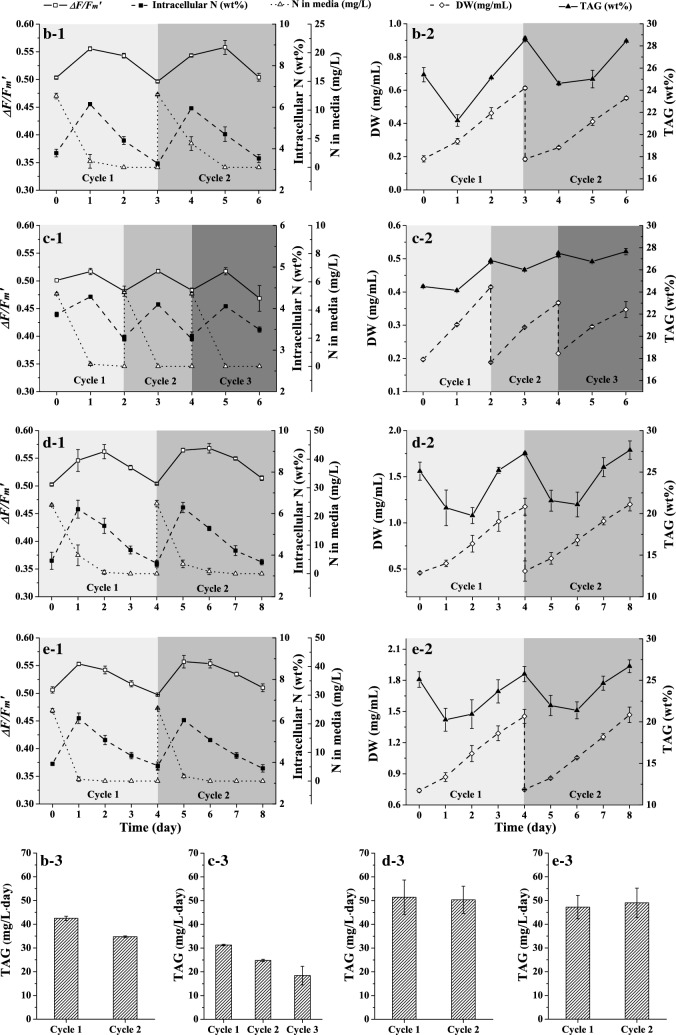
Optimization of the N supply amount and the renewable rate

The proper degree of N-stress has been disclosed for TAG stable production. Using Δ*F/F*_m_*′* as an N-stress indicator, a semi-continuous cultivation of the proper N-stressed day4-cell was established by controlling the N supplying strategy. The initial N concentration in the medium and the renewal rate were optimized as the key operating parameters to improve the preliminary established semi-continuous process since the two operating parameters were reported to greatly affect the specific growth rate of the cells and productivity of the target products in a semi-continuous cultivation [32–34]. The optimization experiments are listed in Table 1, and the results are shown in Figs. 3 and 4. During the cultivation, Δ*F/F*_m_*′* was monitored daily to guide semi-continuous recycling, and the critical point of monitoring for Δ*F/F*_m_*′* was 0.503. When the Δ*F/F*_m_*′* reached the preset critical point of monitoring, a new cycle was restarted. The harvest and additional proportion of medium were the same and were operated according to the renewal rate in Table 1. Additionally, the initial N concentration was adjusted as required in Table 1.

**Table 1:** Operating conditions of five semi-continuous experiments

Experiment	a	b	c	d	e
Renewal rate	0.8	0.8	0.8	0.6	0.3
Initial N concentration (mg/L)	24	12	5	24	24

a to e represents the five experiments

To keep the culture conditions consistent with that of the batch cultivation in “Determination of the proper degree of N-stress of microalgal cells for TAG production” section, in experiment a, the initial N concentration was set to 24 mg/L. The initial cell density and total amount of N, including the intracellular N and free N in the medium, were regulated to the same level as that in the batch cultivation. It should be noted that cycle 1 was started at day 0 and ended at day 4; at the same time, day 4 was also the beginning point of cycle 2, which ended at day 8. The intracellular N at day 0, day 4 and day 8 coincided with each other as shown in Fig. 3a-1. The NSIs were maintained as almost equal, with values of 0.72, 0.71 and 0.70, respectively. The DW and TAG content also showed the same trend between cycles. The results indicated the successful repeat of the proper N-stressed cells defined as day4-cell with an NSI of 0.72 in “Determination of the proper degree of N-stress of microalgal cells for TAG production” section.

Experiments b and c were performed to investigate the effects of the initial N concentration in medium on TAG productivity. As shown in Fig. 4b, c, the TAG productivity of each cycle during experiments b and c were lower than that during experiment a. It is common sense that the DW increment of each cycle is dependent on the total N in the medium. For example, regarding cycle 2 in experiment a, the intracellular N at the end point was set to be equal to the initial intracellular N of 3.82%, and the total N amount added into the medium was fixed to be 22.9 mg/L; thus, theoretically, the DW increment of cycle 2 should be 0.60 mg/mL (the amount of N added into the medium each time divided by the initial intracellular N). Practically, the actual DW increment was 0.65 mg/mL in accordance with the theoretical value. In other words, when the degree of N-stress of the cells at the end point was fixed, which indicated that the intracellular N was set to be at a fixed value, the total N amount added into the medium would decide the absolute increment of DW. Therefore, it was the cell growth rate that actually determined the TAG productivity of each cycle. Additionally, the DW increase rates in experiments b and c were 0.13 and 0.09 mg/mL day, respectively, and were lower than that of experiment a, which was 0.18 mg/mL day. The lower cell growth rate was caused by the lower photosynthetic activity of the cells. In semi-continuous cultivation, the cells used as inoculum were N-stressed with the “sub-health” status. After resupplying the N nutrient, the stressed cells started to recover towards the “health” status. The photosynthetic activity of the cells was recovered as one of the important physiological features. As shown in Fig. 4b-1, c-1, the Δ*F/F*_m_*'* recovered to the peak of 0.55 and 0.52 during each cycle in experiment b and c after N replenishment and recovered to 0.58 in experiment a. Obviously, resupplying the N amount has a large effect on the level of photosynthetic activity recovery. In experiments b and c, the cells did not recover to the health status as in experiment a, leading to the lower cell growth and lower TAG productivity.

**Fig. 5:**
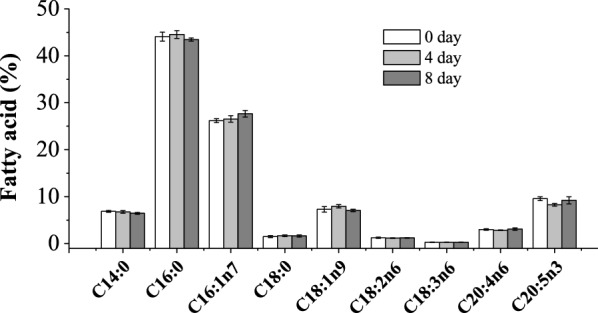
Fatty acid composition of the day4-cell cultured in semi-continuous mode

Another critical operating factor was the renewal rate of the semi-continuous cultivation mode [34]. In this work, three experiments with different renewal rates of 0.8, 0.6 and 0.3 were conducted. Though different from changing the initial N concentration, adjusting the renewal rate obviously improved the stability of TAG productivity. Both the TAG productivity in experiments d and e of each cycle remained at approximately 50 mg/L day, as shown in Fig. 4. Additionally, in Fig. 4, other parameters such as the DW and TAG content showed high reproducibility between cycles. Furthermore, the fatty acid composition of the cells at the beginning and end point of each cycle was analysed. As shown in Fig. 5, after N replenishment at day 0 and day 4 of cultivation, all of the fatty acid compositions were the same at each beginning and end point of the two cycles. The constant fatty acid composition at the end point of each cycle indicated a high consistency of TAG quality. In contrast, the reported semi-continuous cultivation of microalgae under N-stress, which was controlled by other parameters such as biomass concentration or pH et al., showed that the lipid productivity and composition were usually unstable and fluctuated [32, 35, 36].

Compared to the healthy cells with sufficient nutrition, there was an obvious reduction in the photosynthesis activity of the proper N-stressed cells as shown in Fig. 1a, which hindered the cell growth. However, the rate of TAG accumulation of the stressed cells received a tremendous boost instead and remedied the sacrifice of cell growth. Hence, via the semi-continuous cultivation with maintenance of the degree of N-stress by controlling the N supply strategy, stable, sustainable TAG production was achieved.

Taken together, the data indicate that Δ*F/F*_m_*′* is a rapid and in situ N-stress indicator and could precisely guide the control of the degree of N-stress during the cultivation of microalgae for efficient and stable TAG production.

## Conclusion

It is vital to quantify the degree of N-stress when it is applied to obtain maximum TAG productivity during microalgal cultivation. The concept of the nitrogen stress index, NSI, was defined. The photosynthetic activity parameter of microalgae, Δ*F/F*_m_*'*, was identified as a perfect online indicator for the degree of N-stress of the cells. Based on NSI, a novel semi-continuous cultivation strategy of precise N-stress control by the accurate monitoring of Δ*F/F*_m_*'* as an N-stress indicator was established for stable and efficient TAG production. The renewal rate and initial N concentration were optimized as 0.6 and 24 mg/mL, respectively. This new cultivation strategy provides significant guidance for outdoor microaglal cultivation in industrial applications and controlled indoor cultivation.
